# Sero-prevalence and risk factors of hepatitis C virus infection among pregnant women in Bahir Dar city, Northwest Ethiopia: cross sectional study

**DOI:** 10.11604/pamj.2015.21.158.6367

**Published:** 2015-06-25

**Authors:** Yohannes Zenebe, Wondemagegn Mulu, Mulat Yimer, Bayeh Abera

**Affiliations:** 1 Department of Microbiology, Immunology and Parasitology, College of Medicine and Health Sciences, Bahir Dar University, Bahir Dar, Ethiopia

**Keywords:** HCV, pregnant women, Ethiopia

## Abstract

Viral hepatitis during pregnancy is associated with high risk of maternal complications and has become a leading cause of fetal death. So the main objective of this study is to determine the prevalence of hepatitis C viral infections among pregnant women attending the antenatal clinic in Bahir Dar health institutions, Ethiopia. This was institutional based cross-sectional study that included 318 pregnant women who attended the antenatal clinic in Bahir Dar health institutions from January 2013 to June 2013. Appropriate data was gathered from study participants. Sero-prevalence of hepatitis C virus was determined by detecting immunoglobulin of HCV using ELISA kit. Data was entered and analyzed with SPSS version 16 statistical software. The overall prevalence of hepatitis C virus among pregnant women was 0.6%. None of the expected risk factors had significant outcome. In conclusion, prevalence of the Hepatitis C virus among pregnant women attending in Bahir Dar health institutions was low and expected variables were not statistically significant.

## Introduction

Hepatitis viruses are the main cause of liver cancer. Among those, hepatitis C virus is the one that causes hepatocellular carcinoma. It can be ended with development of cirrhosis and hepatocellular carcinoma (HCC). Moreover infection with HBV and HCV affects the liver and results in a broad spectrum of disease outcome [1, 2].

World health organization estimated that approximately 170 million people are infected with HCV, about 130 million are carriers, three to four million persons are newly infected in each year and more than 350,000 people estimated to die from hepatitis C-related liver diseases in each year worldwide [3]. The worldwide prevalence of hepatitis C virus (HCV) infection in pregnant women is estimated to be between 1 and 8% and in children between 0.05% and 5% [4].

Data from the adult population suggest that approximately 10–20% of patients with HCV can go on to develop cirrhosis and hepatocellular carcinoma and the probability of becoming a chronic carrier is inversely related to age at the time of infection [5]. The prevention of vertical transmission is very important, because infection at infancy usually leads to a chronic carrier status [6]. Some of the risk factors for contracting HCV include sharing of injecting equipment, tattooing or piercing with unsterile equipment or procedures, working in environments where there is contact with bodily fluids, receiving blood transfusions, receiving dialysis, surgery, sharing of equipment with a person having HCV, practicing unsafe sex or having contact with blood without adequate protection [7, 8].

Even though some studies on sero-prevalence of HCV infection in Ethiopia have been previously done and indicated as it is endemic in Ethiopia with regional variation [9], no data about the prevalence of hepatitis C virus among pregnant women are available in Bahir Dar city administration. The present study was conducted to determine the prevalence of hepatitis C virus among pregnant women and to identify risk factors associated with hepatitis C virus infection.

## Methods

### Study design, period and area

Institutional based cross sectional study was carried out from January 2013 to June 2013 to assess the sero- prevalence and associated risk factors of Hepatitis C viruses among pregnant women attending antenatal care in Bahir Dar health institutions, Bahir Dar town. Bahir Dar is the capital of the Amhara regional state and situated on the southern shore of Lake Tana, the source of the Blue Nile. It is located approximately 578 km northwest of Addis Ababa, having a latitude and longitude of 11°36'N 37°23'E Coordinates and an elevation of 1840 meters above sea level. Based on the 2007 Census conducted by the Central Statistical Agency of Ethiopia (CSA), Bahir Dar Special Zone has a total population of 221,991, of whom 108,456 are men and 113,535 women; 180,174 or 81.16% are urban inhabitants, the rest of population are living at rural kebeles around Bahir Dar. From the town there is one governmental referral hospital (FelegeHiwot hospital) and 10 governmental health centers. The hospital and 3 randomly selected health centers (Bahir Dar health centers, Han health centers and Tis Abay health centers) were included as our study sites.

### Population and sample size determination

The source population was all pregnant women who have access to attend antenatal care from Bahir Dar health institutions, whereas the study population was all pregnant women who attended antenatal care from the selected health institutions during the study period. The study subjects were also all pregnant women who attended antenatal care at the selected health institutions during data collection time that fulfill the inclusion criteria and signed the consent form. The single population proportion formula was used to determine the sample size and accordingly 318 study subjects were included in this study. The number of sample size in each of the selected study sites was proportionally allocated and convenient sampling technique was used until the sample size completed.

### Data collection

#### Administration of questioners

A pretested structured questionnaire was used to collect socio-demographic characteristics and other expected risk factors of the clients. The data collectors were trained laboratory technologist/technician and nurses.

#### Sample collection and laboratory investigation

About 5ml of whole blood sample was drawn through vein-puncture, and serum was separated and stored in a refrigerator in the study area. The collected samples from the study sites was taken into the place where the laboratory investigation takes place. Sero-prevalence of hepatitis C virus was determined by detecting immunoglobulin of HCV using ELISA kit based on the manufacturer's instruction. Positive results were retested again with ELISA.

#### Quality control

The questionnaire was pretested at the health center other than the actual study sites. The collected data was daily checked for consistency and accuracy. Standardized procedures was strictly followed at each of pre-analytical, analytical and post analytical process. Positive and negative controls were run alongside the test.

#### Data processing and analysis

Data was entered and analyzed using SPSS Version 16.0 statistical software. Binary logistic regression was done to determine the presence of a statistically significant association between explanatory variables and the outcome variables. Odds Ratio (OR), p-value and their 95% Confidence Intervals (CI) was calculated.

#### Ethical considerations

The study was conducted after obtaining institutional ethical clearance from ethical review committee of College of Medicine and Health Sciences, Bahir Dar University. Verbal consent was also obtained from the study participants. Positive results was reported to their physicians for the treatment and any antenatal care. Confidentiality was maintained and the clinical specimen collected during the study period was used for the stated objectives only.

## Results

### Demographic characteristics

A total of 318 pregnant women was included in this study. The mean age of the study participants was 25.72 (SD. ±5.14) years old. Among the total number of participants, 214 (67.3%) were living in an urban setting. The majority of the study participants were housewives (56.9%). One hundred twenty five (39.3%) of the participants did not attend formal education.

### Prevalence of HCV

Among 318 pregnant women, 2 (0.6%) were confirmed as HCV positive. All of HCV positive pregnant women were among orthodox religion followers, married and on secondary trimester. None of the expected risk factors (history of blood transfusion, dental manipulations, tattooing circumcision etc. and other socio-demographic factors) had been found to be associated with HCV positivity (Table 1).

**Table 1:** Prevalence of HCV among pregnant women attending antenatal care in Bahir Dar city adminstration, 2013 (n = 318)

Socio-demographic variables	HCV	
	Positive, N (%)	Negative, N (%)
**Residence**		
Urban	1 (0.5)	213 (99.5)
Rural	1 (1.0)	103 (95.2)
**Age groups (yrs.)**		
**<21**	0 (0.0)	81 (100.0)
21-24	0 (0.0)	84 (100.0)
25-30	1 (0.9)	108 (99.9)
>30	1 (2.3)	43 (97.7)
**Religion**		
Orthodox	2 (0.7)	272 (99.3)
Muslim	0 (0.0)	40 (100.0)
Protestant	0 (0.0)	4 (100.0)
**Occupation**		
Merchant	1 (2.4)	41 (97.6)
Student	0 (0.0)	18 (100.0)
Housewife	1 (0.6)	180 (99.4)
Day laborer	0 (0.0)	16 (100.0)
Employer	0 (0.0)	35 (100.0)
Others	0 (0.0)	26 (100.0)
**Educational status**		
Not literate	1 (0.8)	124 (99.2)
Primary level	0 (0.0)	79 (100.0)
Secondary level	0 (0.0)	59 (100.0)
Diploma and above	1 (1.8)	54 (98.2)
**Marital status**		
Unmarried	0 (0.0)	15 (100.0)
Married	2 (0.7)	294 (99.3)
Divorced	0 (0.0)	6 (100.0)
Widowed	0 (0.0)	1 (100.0)
**Average monthly income (Birr)**		
<1000	1 (0.8)	118 (99.2)
1000-1500	0 (0.0)	64 (100.0)
1501-2300	0 (0.0)	58 (100.0)
>2300	1 (1.3)	76 (98.7)
**Pregnancy stage**		
1° trimester	0 (0.0)	60 (100.0)
2° trimester	2 (1.4)	139 (98.6)
3° trimester	0 (0.0)	117 (100.0)

## Discussion

Infections due to Hepatitis C viruses (HCV) is significant health problems around the globe. Worldwide, viral hepatitis is the commonest cause of hepatic dysfunction in pregnancy. In our study, the frequency of Hepatitis C virus infections among pregnant women attending antenatal care in Bahir Dar health institutions was 0.6%. This was similar to the study which was conducted from Sudan, (0.6%) [10]. All of the HCV positive pregnant women were among secondary trimesters and orthodox religion followers.

In contrast the prevalence of HCV in our finding was less than the studies conducted from Nigeria (3.6%) [11], Cameron (1.9%) [12], Egypt (6.4%) [13] and Gondar, Ethiopia (1.3%) [14]. These discrepancies might not be disparate with the fact that some of the studies were not from the same risk group and some of the them were done with the detection of both hepatitis C virus RNA and anti HCV antibody, which was Anti HCV antibody detection only in case of our study. This may loss the acute infections before antibody production.

None of the expected risk factors (history of blood transfusion, sugery, dental manipulations, tattooing circumcision etc. and other socio-demographic factors) for sero-positivity of HCV had been identified in the study. This might be due to the small sample size we used. Moreover in similar studies reported at Nigeria and Sudan, these expected risk factors were not associated for the positivity of HCV [10, 11]. The explanations for such observations need to be addressed in the future.

## Conclusion

The frequency of hepatitis C virus in this study was low. None of the expected risk factors for sero-positivity of HCV had been also identified in the study. There is a need of further study on large sample size and using both antibody and RNA detection of HCV.
